# BK Polyomavirus Infection of Bladder Microvascular Endothelial Cells Leads to the Activation of the cGAS‐STING Pathway

**DOI:** 10.1002/jmv.70038

**Published:** 2024-11-02

**Authors:** Kateřina Bruštíková, Boris Ryabchenko, David Liebl, Lenka Horníková, Jitka Forstová, Sandra Huérfano

**Affiliations:** ^1^ Department of Genetics and Microbiology, Faculty of Science Charles University, BIOCEV Vestec Czech Republic; ^2^ Imaging Methods, Core Facility, Faculty of Science Charles University, BIOCEV Vestec Czech Republic

**Keywords:** BK polyomavirus, BKPyV reservoir cells, cGAS, interferon response, STING

## Abstract

BK polyomavirus (BKPyV) infection in humans is usually asymptomatic but ultimately results in viral persistence. In immunocompromised hosts, virus reactivation can lead to nephropathy or hemorrhagic cystitis. The urinary tract serves as a silent reservoir for the virus. Recently, it has been demonstrated that human bladder microvascular endothelial cells (HBMVECs) serve as viral reservoirs, given their unique response to infection, which involves interferon (IFN) production. The aim of the present study was to better understand the life cycle of BKPyV in HBMVECs, uncover the molecular pathway leading to IFN production, and to identify the connection between the viral life cycle and the activation of the IFN response. Here, in the early stage of infection, BKPyV virions were found in internalized monopinocytic vesicles, while later they were detected in late endosomes, lysosomes, tubuloreticular structures, and vacuole‐like vesicles. The production of viral progeny in these cells started at 36 h postinfection. Increased cell membrane permeability and peaks of virion release coincided with the leakage of viral and cellular DNA into the cytosol at approximately 60 h postinfection. Leaked DNA colocalized with and activated cGAS, leading to the activation of STING and the consequent transcription of *IFNB* and IFN‐related genes; in contrast, the IFN response was attenuated by exposure to the cGAS inhibitor, G140. These findings highlight the importance of the cGAS‐STING pathway in the innate immune response of HBMVECs to BKPyV.

## 1 Introduction

Polyomaviruses (PyVs) are small, double‐stranded (ds) DNA viruses found widely in nature. To date, 13 human PyVs have been identified [1, 2]. While these viruses can be found in healthy populations, they have also been associated with serious diseases in immunocompromised patients. The most important human PyVs are BKPyV, which is associated with nephropathy and hemorrhagic cystitis; JCPyV, responsible for progressive multifocal leukoencephalopathy; and Merkel cell carcinoma PyV, which is involved in the development of skin cancer.

The worldwide prevalence of BKPyV is remarkably high, as evidenced by serological studies. Approximately 80% of adults have been reported to be seropositive for BKPyV [3, 4, 5, 6]. Following primary asymptomatic infection, the virus disseminates via the bloodstream and establishes persistence in the urinary tract. This persistence is linked to low‐level viral production [7, 8]. The reactivation of viral infection after kidney transplantation can result in nephritis [9, 10] or graft loss [11]. Similarly, in bone marrow transplant recipients, BKPyV infection can result in hemorrhagic cystitis or nephritis [7]. Currently, there are no specific antiviral treatments available for BKPyV infection.

BKPyV is a non‐enveloped virus with an isometric capsid of approximately 40 nm in diameter containing a circular dsDNA genome (approximately 5 kb long) complexed with host cell histones. Polyomaviruses replicate in host cell nuclei and their genomes encode a limited number of multifunctional proteins. The early region of primate PyV encodes the small tumor (sT) and large tumor (LT) antigens, while the late region encodes the capsid proteins VP1, VP2, and VP3 and a late auxiliary protein known as agnoprotein [12].

Initial studies on BKPyV were carried out using monkey epithelial cells (Vero); in subsequent years, however, experiments were extended to human renal proximal tubular epithelial cells (RPTECs), which are the cells targeted by BKPyV during its reactivation [13]. The virus follows similar entry and trafficking pathways in all the cell types investigated. BKPyV binds to beta‐series gangliosides GD1b and GT1b via an α2,8‐linked sialic acid recognition motif [14, 15, 16]; then, is internalized into monopinocytic vesicles derived from lipid rafts, via caveolae dependent or independent mechanisms [17, 18, 19]. Once inside the cell, the virus passes through the endosomal sorting, eventually reaching the endoplasmic reticulum (ER), bypassing the Golgi apparatus [18, 20].

Approximately 6–10 h postinfection (hpi), BKPyV reaches the ER [18, 21], where virions undergo rearrangements. Studies employing model polyomaviruses (simian SV40 and mouse polyomavirus [MPyV]) have demonstrated that changes in the capsid such as the reduction and isomerization of disulfide bonds, result in the exposure of the hydrophobic minor proteins (VP2 and VP3) on the capsid surface. The “hydrophobic” particles then interact with ER membranes, thereby facilitating viral exit into the cytosol [22, 23, 24, 25, 26]. Consistent with this, the exposure of BKPyV VP2/VP3 during cell entry was later demonstrated [27]. Following cytosolic translocation, the nuclear localization signals of the PyVs capsid proteins interact with importins, allowing the entry of the virus into the nucleus and, consequently, virus replication [27, 28, 29].

Interestingly, while most non‐enveloped viruses release their progeny primarily after cell lysis, BKPyV, uses an additional active mechanism yet poorly understood to exit the cells which involves vesicular trafficking and accounts for the release of a small percentage of the virus [30, 31].

Several studies have demonstrated that RPTECs are immunologically nonresponsive to BKPyV infection [32, 33, 34]. In contrast, other cells within the urinary tract, specifically human bladder microvascular endothelial cells (HBMVECs), activate the interferon (IFN) type I pathway in response to BKPyV infection [33]. These immune‐responsive cells would therefore serve as a putative viral reservoir, allowing the virus to replicate at low levels and thus persist.

Viruses activate but also strategically modulate IFN responses of the host cells to promote their replication. Recently, various DNA viruses including MPyV DNA have been reported to induce the activation of IFN pathways via cyclic guanosine‐adenosine synthetase (cGAS) [35, 36]. cGAS is a key sensor of viral DNA in cytosol. Mechanistically, upon binding to DNA, cGAS catalyzes the synthesis of 2′3′‐cyclic guanosine‐adenosine monophosphate (cGAMP) [37]. The cGAMP molecule binds to cytosolic binding ligand domain of the stimulator of interferon genes (STING), an ER transmembrane protein, which triggers STING oligomerization and its translocation from the ER to Golgi‐perinuclear areas. In the Golgi, STING interacts with TANK‐binding kinase 1 (TBK1), leading to its phosphorylation and the subsequent recruitment and phosphorylation of interferon regulatory factor 3 (IRF3). The nuclear factor kappa‐light‐chain‐enhancer of activated B cells (NF‐κB) can be also activated, although how this occurs remains controversial. IRF3 and NF‐κB enter the nucleus and activate IFNB transcription which then leads to the induction of interferon‐stimulated genes (ISGs) such as *ISG15*, *ISG56*/*IFIT1,* and chemokines, for example, *CXCL10*. Interferon‐induced proteins limit viral replication and spread [37, 38]. The cGAS‐STING pathway plays a critical role in mediating immune defences against DNA viruses.

The aim of this study was to unravel the molecular details of BKPyV replication, including kinetics of the BKPyV life cycle and the mechanisms underlying the activation of the IFN response in primary HBMVECs, with a particular focus on the role of cGAS‐STING pathway.

## 2 Results

### 2.1 The BKPyV Life Cycle in HBMVECs

**Figure 1:**
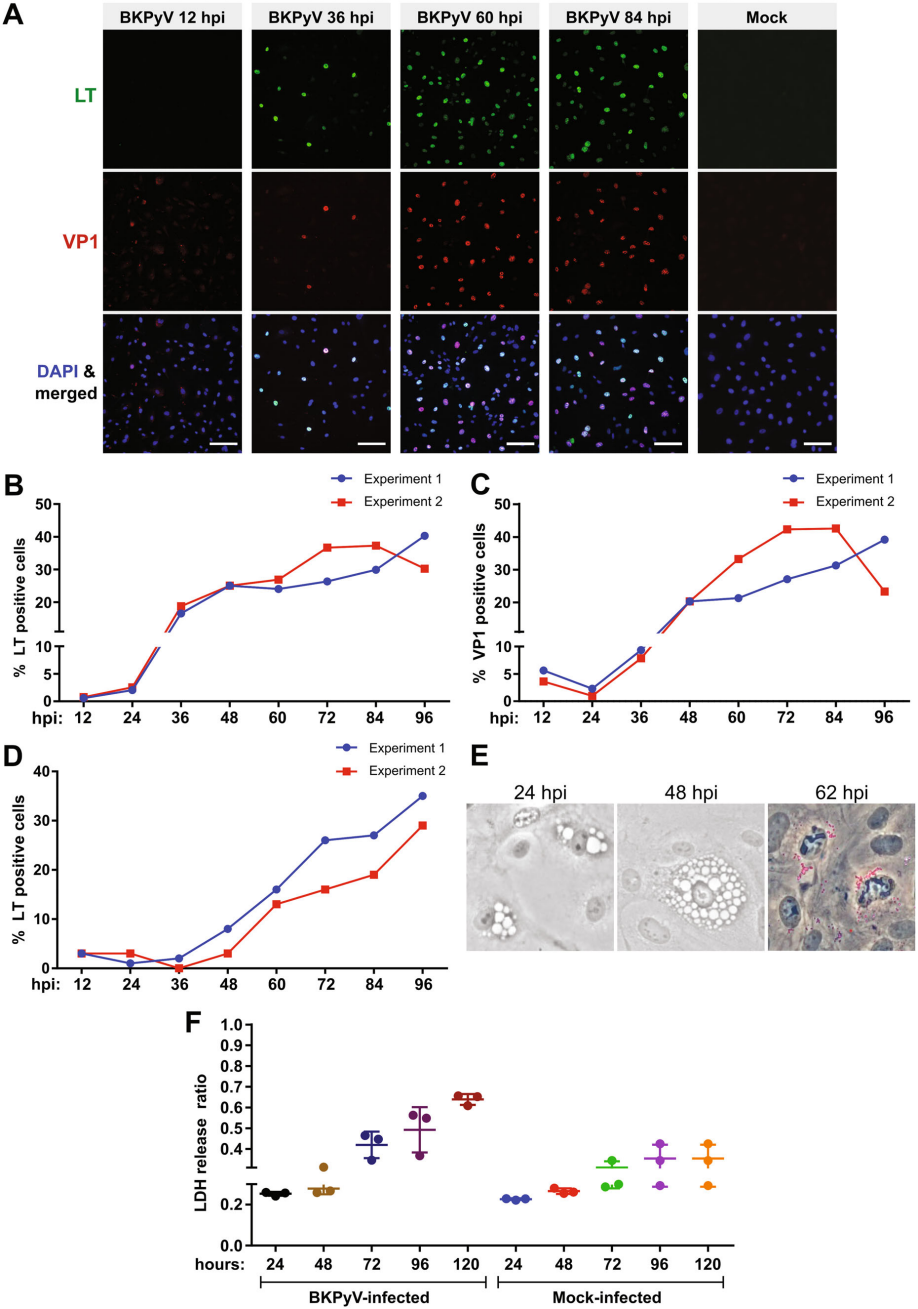
The kinetics of BK polyomavirus (BKPyV) infection in human bladder microvascular epithelial cells (HBMVECs). Cells were infected at MOI of 0.5 FFUs/cell (A–E), at MOI of 10 FFUs/cell or mock‐infected (F). At the indicated times, the cells were fixed on coverslips, or medium was collected. (A–C) The percentage of cells expressing the viral antigens was evaluated in two independent experiments. Cells were stained using antibodies against LT antigen (green) or VP1 protein (red); DNA was counterstained with Hoechst 33342 (blue). (A) Representative fields of infected cells at 12, 36, 60, and 84 hpi are shown. As a control, mock‐infected and stained cells are presented. Scale bars correspond to 100 µm. (B, C) Fluorescence images were analyzed using a standardized image analysis pipeline. To determine the percentage of LT‐positive (B) or VP1‐positive (C) cells, 20 000 cells were scored. (D) Quantification of the release of new progeny. At the indicated times after infection, medium from infected cells was collected and used to infect HBMVECs grown on coverslips. At 48 hpi, immunostaining with an anti‐LT antigen–antibody was performed to calculate the percentage of infected cells, with 450 cells being scored at each time point. (E) Cell morphology was assessed by bright‐field (BF) microscopy at 24, 48, and 62 hpi. At 62 hpi a stain for detection of neutral lipids (red) was used. (F) The LDH release ratio was determined by measuring the spontaneous release in supernatants and the total level of LDH release after treatment with Triton. Three experiments were conducted and means ± SD are presented in the graph.

The present study is the second approach to the use of HBMVECs to study BKPyV. Primary HBMVECs were used to explore the kinetics of the BKPyV life cycle. Notably, given the challenges associated with primary cell propagation and maintenance, the cells were not synchronized for the experiments. Cells were infected with BKPyV at a multiplicity of infection (MOI) of 0.5 fluorescent focus‐forming units (FFUs) per cell. In two independent experiments, viral protein production was monitored by immunofluorescence over 4 days (analyzed in 12 h intervals). Representative fields of infected or mock‐infected cells stained for LT antigen, an early protein, and VP1, a late major capsid protein, are shown in Figure 1A. The percentages of cells expressing LT and VP1 were calculated from these microscopic fields (Figures 1B,C, respectively) using a standardized fluorescence image analysis assay. In this analysis, only cells with LT‐positive and VP1‐positive nuclei were counted, respectively.

LT antigen expression was first detected at 12 hpi in a tiny cell subpopulation. Subsequently, there was an accelerated increase in the number of LT‐positive cells up to 48 hpi, reaching an initial peak of approximately 25%, followed by a steady increase until 84 hpi for Experiment 2 and 96 hpi for Experiment 1 (Figure 1B).

Although VP1‐positive cells were detected at 12 and 24 hpi, the positivity at these time points can be explained by: (i) the presence of incoming virus in the cytoplasm close to the nucleus, which led to these cells being analyzed by the computer as positive (false positives), and/or (ii) at 24 hpi, a few cells already expressing VP1 could have been detected. At 36 hpi, newly produced VP1 was detected in the nucleus. As expected, VP1 production was delayed compared with that for LT; at 36 hpi, the percentage of LT‐positive cells was twice that of VP1. Subsequently, the percentage of VP1‐positive cells gradually increased until 84 and 96 hpi for Experiment 2 and Experiment 1, respectively (Figure 1C).

In addition to follow the kinetics of the BKPyV life cycle within these cells, the production of new viral progeny was quantified. For this, an infectivity assay was performed using medium from infected cells collected at the indicated times (Figure 1D). In medium from cells infected for 48 h, only low levels of the virus were detected; however, the number of viruses released increased markedly from 60 hpi (Figure 1D).

To follow cell death induced by the BKPyV infection, the permeabilization of plasma membrane was then assessed by the release of lactate dehydrogenase (LDH) from cells. A high MOI (10 FFUs/cell) was used for infection to allow for better visualization of toxicity. Notably, although the cells displayed vacuole‐like vesicles and lipid droplet deposition (see more details in Supporting Information S1: Figure 1) soon after infection, suggestive of cytotoxicity (Figure 1E), the release of LDH from infected cells was detected only at 72 hpi. Interestingly, at this time point, the LDH levels were only approximately 20% higher than those spontaneously released by noninfected cells. Toxicity gradually increased from 72 to 120 hpi, the last sampling time point (Figure 1F).

To follow closely some of the events occurring during BKPyV infection in HBMVECs, the subcellular localization of viral proteins and virions at different points of the life cycle was monitored.

**Figure 2:**
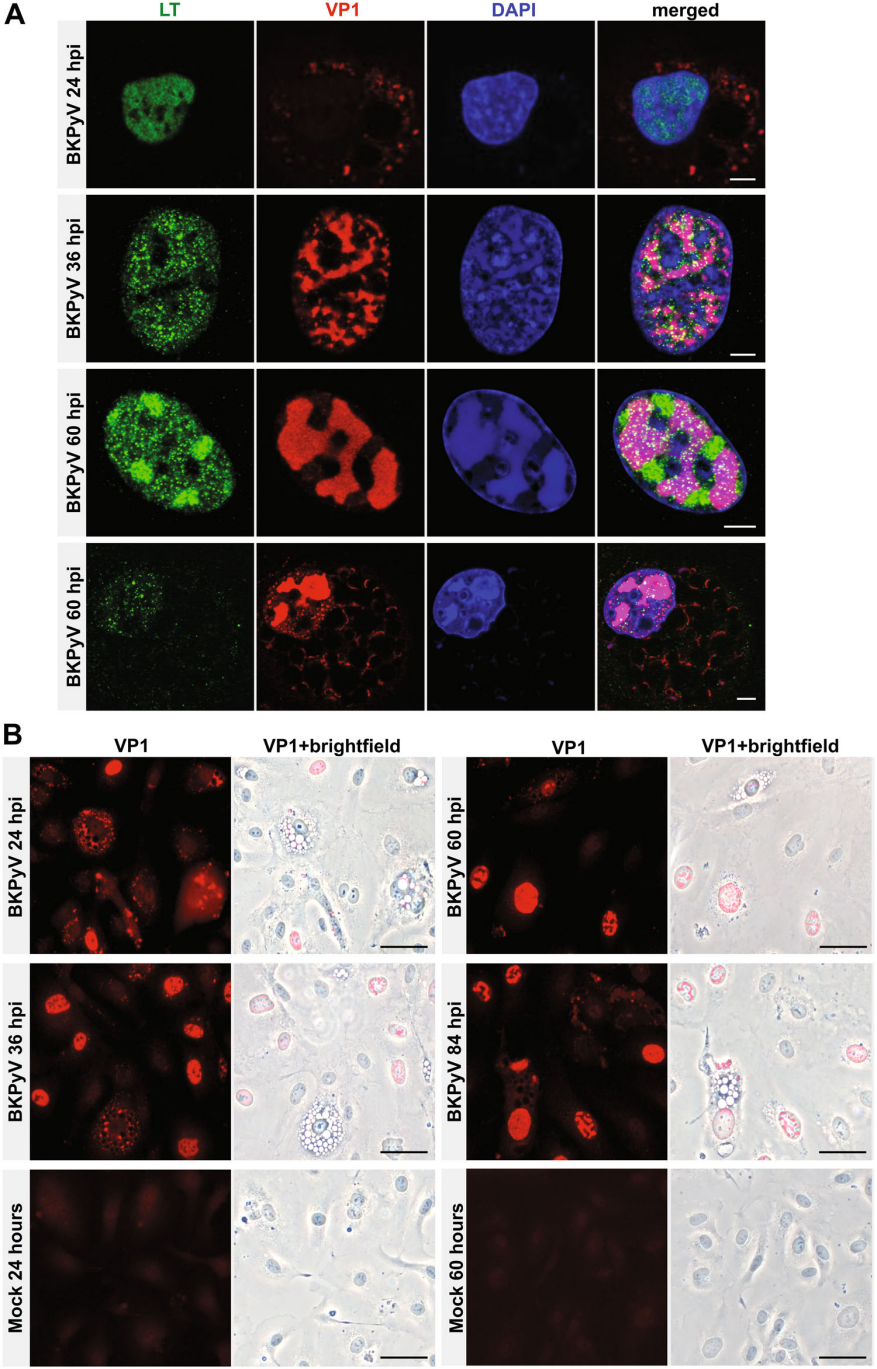
Analysis of subcellular localization of viral proteins during BK polyomavirus (BKPyV) infection of human bladder microvascular endothelial cells (HBMVECs). Primary HBMVECs were infected with BKPyV at MOI of 20 FFUs/cell, fixed on coverslips. (A) cells were stained using antibodies against the LT (green) or VP1 (red) protein. DNA was stained with Hoechst 33342 (blue). Representative confocal images of cells at 24, 36, and 60 hpi are shown. Scale bars correspond to 10 µm. (B) Cells were stained at 24, 36, 60, and 84 hpi and subjected to immunofluorescence staining for the late protein VP1 (red; left panels). In addition, in these cells, the presence of vacuole‐like vesicles (bright field) was analyzed by widefield microscopy (right panels). As a control, mock‐infected and stained cells are presented. Scale bars correspond to 50 μm.

First, HBMVECs cells were infected with the virus at an MOI of 20 FFUs/cell and processed at the indicated time points for the analysis of LT or VP1 protein localization using confocal microscopy and widefield microscopy (Figure 2).

At 24 hpi, the VP1 signal was detected as foci or clusters in the cytoplasm (Figure 2A), in some cells, it was associated with vacuole‐like vesicles, which were observed in approximately 13% of the cells (Figure 2B). The VP1 signal may represent virions (intact or partially disassembled) or newly synthesized VP1 (as the polyclonal antibody used recognizes VP1 in intact virions or VP1 protein newly synthesized). At 24 hpi the early LT antigen expression was already detectable (Figure 2A). From 36 hpi, VP1 was detected in the nucleus (likely new production), primarily near the LT antigen (Figure 2A). At 60 hpi (Figure 2A), the LT antigen was often organized in clusters spatially separated from the VP1 signal. This organization likely corresponds to the compartmentalization of replication and virus assembly centers. Interestingly, another phenotype was also observed in a small subpopulation of cells (Figure 2A, 60 h, lower panel), in these cells, a substantial VP1 signal was found in the cytoplasm, following the pattern of the vacuole‐like vesicles, while LT signal was already low. This pattern suggests that LT signal is cleared at late times postinfection. The spatiotemporal organization of the proteins is consistent with the kinetics of the BKPyV infection (Figure 1). On the other hand, the presence of vacuole‐like vesicles during the early and late times postinfection near to the VP1 signal was intriguing and therefore an ultrastructural analysis was carried out below.

**Figure 3:**
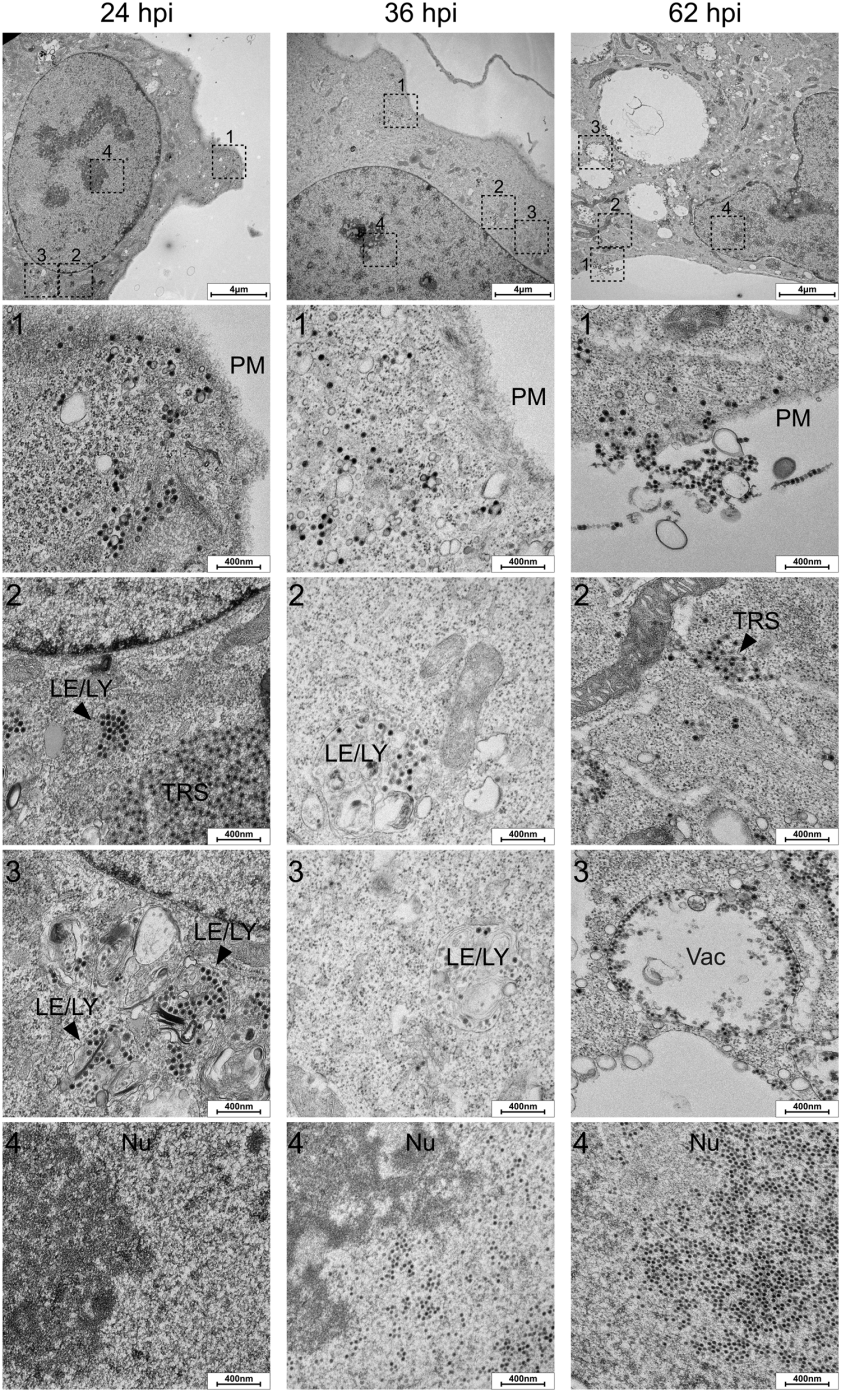
Electron microscopic analysis of the BK polyomavirus (BKPyV) infectious cycle in human bladder microvascular endothelial cells (HBMVECs). Primary HBMVECs were infected with BKPyV at MOI of 20 FFUs/cell; at the indicated times, the cells were fixed and processed for electron microscopy. The figure shows representative images of sections through cells (top panel), with enlarged subcellular areas (1, 2, 3, 4). The compartments in which the virions were detected include the plasma membrane (PM), late endosomes (LE), lysosomes (LY), tuboloreticular structures (TRS), vacuole‐like vesicles (Vac), and nucleus (Nu).

To follow the localization of virions in the subcellular compartments, cells were infected with the virus at an MOI of 20 FFUs/cell and processed at 24, 36, and 62 hpi for ultrastructural analysis by electron microscopy (Figure 3).

The electron microscopy data (Figure 3) showed that at 24 hpi most of the virus was localized at the cell periphery (plasma membrane and smooth, uncoated, caveolae‐like invaginations) and within internalized monopinocytic vesicles; however, a substantial number of virions were also detected within intracellular membranous compartments (late endosomes, lysosomes, and tubuloreticular structures [TRSs]), mostly with perinuclear localization. No progeny virions were detected in the nucleus. At 36 hpi, most virions had been cleared from the cell periphery but were still readily detectable in cytoplasmic membranous compartments. Importantly, progeny virions were already detected in the nuclei of infected cells. At the late‐stage postinfection (62 h), progeny virions accumulated in large clusters in the nuclei of infected cells, but the virus was also ubiquitous in large cytoplasmic vacuole‐like vesicles, cytoplasmatic intracellular membranous compartments (endosomes or TRSs), so as at the cell surface, either associated with the plasma membrane or in monopinocytic vesicles. However, it was not possible to discriminate whether these were progeny virions produced by and released from infected cells, or whether they represented reinfection by virions originating from surrounding infected cells.

The above data indicate that the virus gains entry into the HBMVECs through endocytosis, passes through endosomal compartments and subsequently reaches the nucleus. LT production is detected at 12 hpi, followed by the appearance of new viral progeny at 36 hpi. Notably, a significant release of viral progeny starts at approximately 60–72 hpi, coinciding with a marked increase in cell membrane permeability. Considering that the entire cell population did not die simultaneously, the complete BKPyV life cycle in HBMVECs lasted approximately 3–4 days. The life cycle of BKPyV in HBMVECs described here is similar to the life cycle described for the virus in RPTECs [18, 19, 39], but the presence of the vacuole‐like vesicles containing virions, the presence of lipid droplets, and the frequently observed presence of virus‐loaded TRS at early times postinfection are described here as novels features of the BKPyV infection.

### 2.2 The Mechanism Involved in the Innate Immune Response of HBMVECs to BKPyV Infection

**Figure 4:**
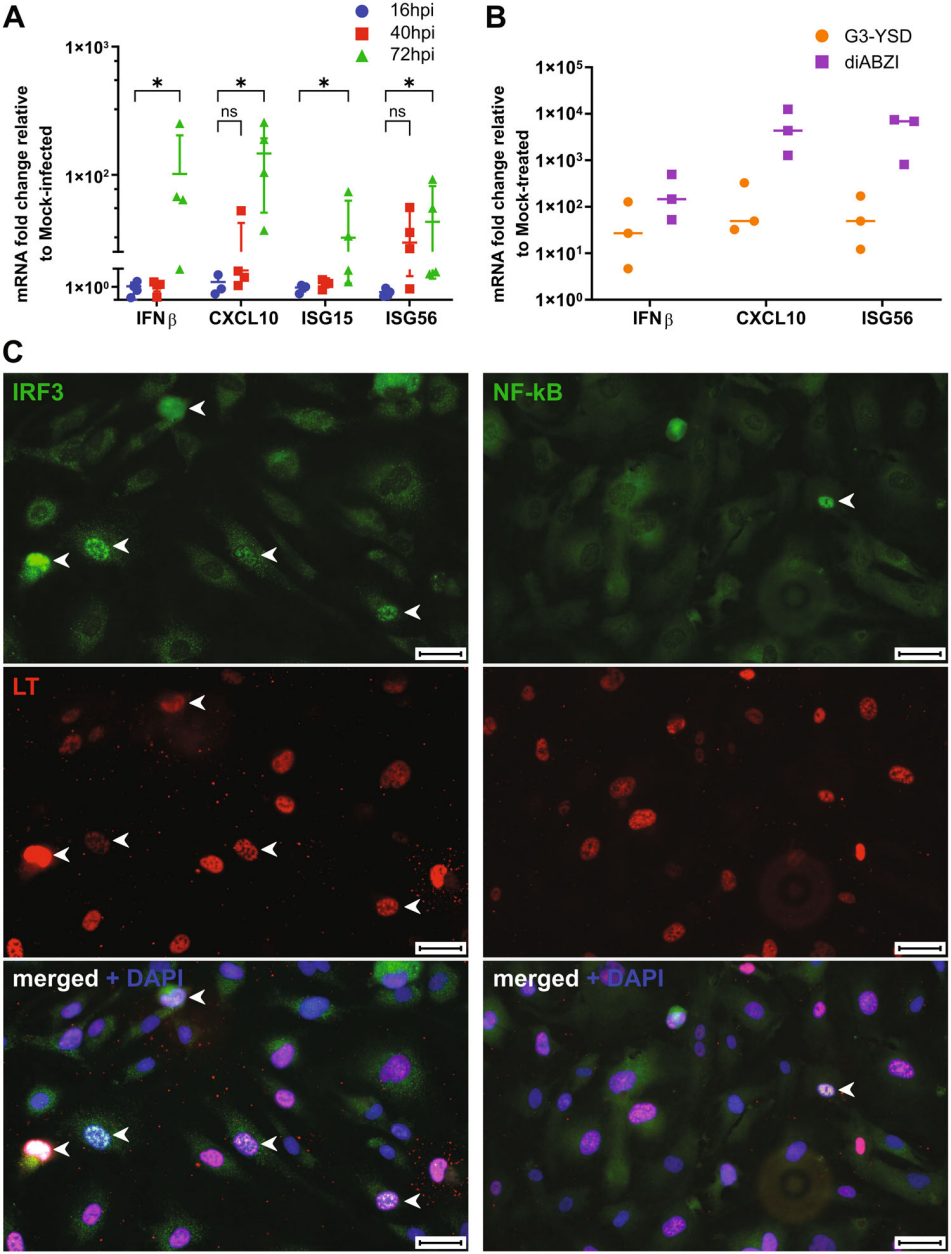
BK polyomavirus (BKPyV) infection induces the IFN response in primary human bladder microvascular endothelial cells (HBMVECs). (A) BKPyV‐infected at MOI of 10 FFUs/cell and mock‐infected HBMVECs were harvested at indicated times for RT‐qPCR. For each sample, the mRNA levels of *IFNB*, *CXCL10*, *ISG56*, and *ISG15* were normalized to those of *GAPDH*, and the fold increase was obtained by comparing each sample to the mock‐infected cells corresponding to each time point. Data represent the fold increase of fourth independent experiments. Statistics were calculated using GraphPad Prism software version 10. Probability levels are indicated by n.s. (not significant) and *p ≤ 0.05, Mann–Whitney *U* test. (B) Cells were transfected with G3‐YSD or treated with diABZI and harvested after 16 h for RT‐qPCR. The expression levels of *IFNB*, *CXCL10*, and *ISG56* were normalized to those of *GAPDH* and the fold increase was obtained by comparing each sample to the mock‐transfected cells (Turbofect‐for G3‐YSD) or untreated cells (for diABZI). The data represent three independent experiments. (C) Cells infected with BKPyV were fixed at 62 hpi and stained with antibodies against IRF3 or NF‐κB (green), LT (red), and counterstained with DAPI. Representative fields are displayed. The arrows indicate the subpopulation of cells with the proteins in the nucleus. Scale bars correspond to 50 μm.

It has been shown that HBMVECs launch an IFN response following BKPyV infection [33]. Here, primary HBMVECs were infected with BKPyV at a high MOI (10 FFUs/cell) to closely monitor the kinetics of the IFN response and identify the underlying mechanism. The MOI 10 was chosen based on pilot experiments that showed that the upregulation of IFN is MOI dependent (data not shown). The kinetics of the IFN response was assessed at 24, 40, and 72 hpi by determining the expression levels of *IFNB* and the IFN‐inducible genes *ISG15*, *ISG56*/*IFIT1*, and *CXCL10* (Figure 4A). The results confirmed that the IFN response was robustly established by 72 hpi, characterized by upregulation of *IFNB*, *CXCL10*, and *ISG56* expression. However, increased levels of *ISG56* and *CXCL10*, transcripts were detected at 40 hpi, at this time point, this response is likely IFN‐independent as no upregulation of the expression of *IFNB* expression was detected at this time. To assess the ability of the cells to respond to other stimuli, the cells were transfected with G3‐YSD, a 26‐mer DNA that specifically activates cGAS, and amido benzimidazole (diABZI), a STING agonist (Figure 4B). Both stimuli induced the transcription of *IFNB* and IFN‐associated genes in HBMVECs. Notably, the IFN response was higher with diABZI than with the cGAS agonist. To better understand the magnitude of the IFN response, the nuclear translocation of IRF3 and NF‐κB from the cytosol was examined at 62 hpi (Figure 4C). IRF3 was detected in the nucleus in 11% of the infected cells, whereas NF‐κB translocation was only sporadically observed (1% of the infected cells).

Overall, the data show that the key event underlying the stimulation of the IFN response during BKPyV infection of HBMVECs occurred late postinfection. The magnitude of this IFN response was similar to that induced by the cGAS agonist. Importantly, the IFN response to BKPyV was largely due to the activation of the IRF3 transcription factor.

### 2.3 Analysis of the cGAS‐STING Pathway

**Figure 5:**
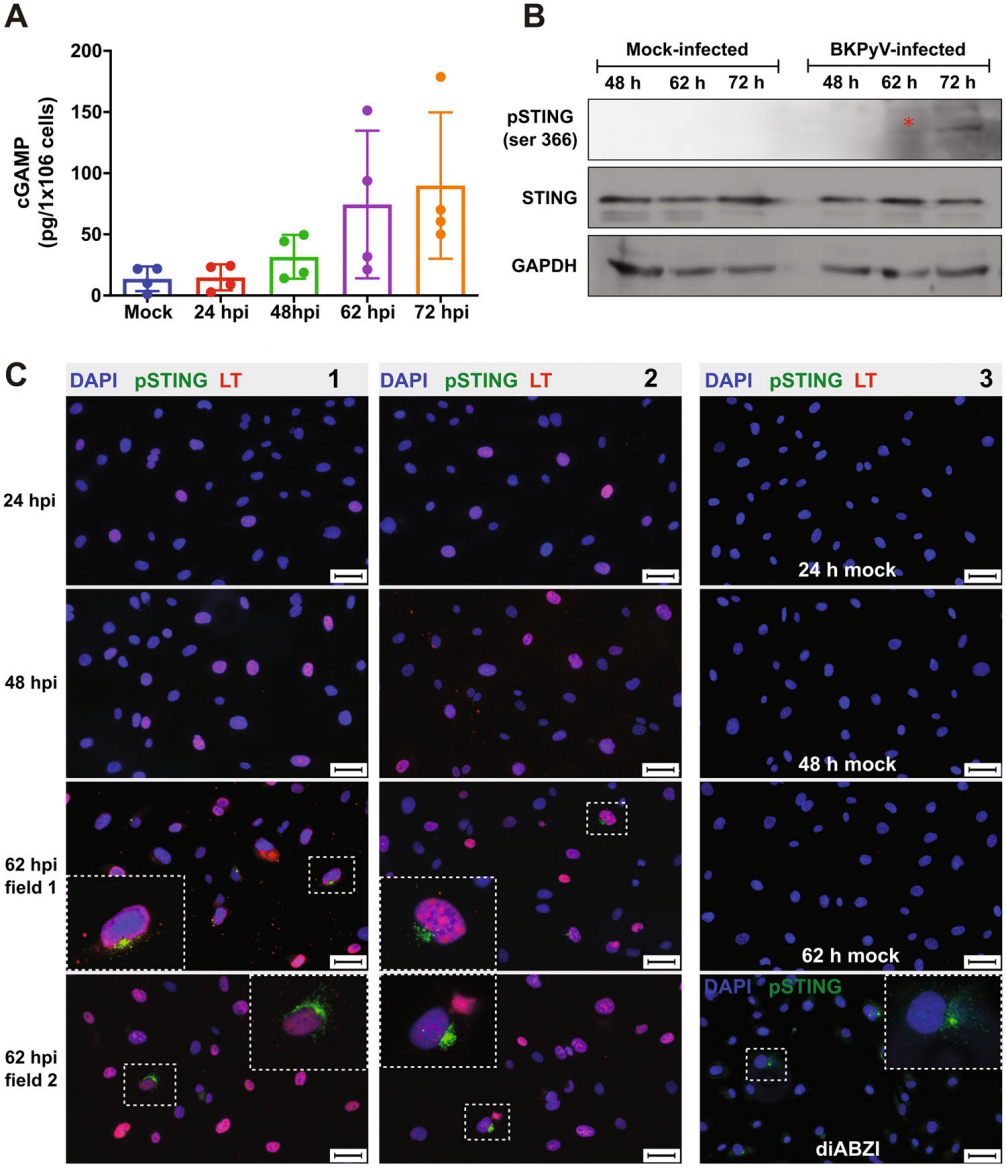
The cGAS‐STING pathway is activated in human bladder microvascular endothelial cells (HBMVECs) during BK polyomavirus (BKPyV) infection. Cells infected with BKPyV at MOI 10 FFUs/cell were harvested to prepare cell lysates or fixed at the indicated times. (A) cGAMP levels were measured in cell lysates by competitive enzyme‐linked immunosorbent assay (ELISA). The graph shows the mean values ± the standard deviation of four independent experiments for each time point. (B) Western blot analysis for the detection of phospho‐STING and, as controls, STING and GAPDH in BKPyV‐infected or mock‐infected cells. The image shows one of two independent experiments. A star (*) was used to highlight low levels of the protein. (C) Cells infected with BKPyV, treated with diABZI, or mock‐treated were fixed and stained with antibodies against phospho‐STING (green) and LT (red), DNA was stained with DAPI (blue). Two independent experiments were performed and representative fields from both experiments are presented (panels 1 and 2, respectively). Mock‐infected cells or cells treated with diABZI, a STING agonist, were included as controls (Panel 3). Selected cells (dotted white boxes) are magnified and displayed (bigger dotted white boxes). Scale bars correspond to 50 µm.

To elucidate the molecular mechanism underlying the activation of the IFN response, the cGAS‐STING pathway was investigated. The binding of DNA to cGAS induces the formation of protein foci through liquid phase separation (cGAS liquid‐like droplets) in which the sensor is activated, leading to the production of 2′‐3′cGAMP [40]; accordingly, the production of cGAMP at different times postinfection (24, 48, 62, and 72 hpi) was assessed using whole cell lysates in four independent experiments. Competitive enzyme‐linked immunosorbent assay (ELISA) revealed that cGAMP production (above the levels of the mock cells) occurred between 48 and 72 hpi (Figure 5A). Specifically, cGAMP was detected at 48 hpi in two out of the four experiments and by 72 hpi, all samples were positive for cGAMP. The fluctuation in cGAMP levels may be attributed to asynchronous infection dynamics within the cell population. cGAMP was not detected in the supernatant (medium) of the infected cells (data not shown).

Subsequently, the levels of STING phosphorylation were evaluated by western blot at 48, 62, and 72 hpi. Very low levels of phospho‐STING were found at 62 hpi and increasing at 72 hpi (Figure 5B). To better discern the subpopulation of cells expressing phospho‐STING, immunofluorescence was performed on cells fixed at 24, 48, and 62 hpi (Figure 5C). A subpopulation of cells was identified that display phospho‐STING only from 62 hpi (Figure 5C: Experiment 1 [panel 1] and Experiment 2 [panel 2]). Mock‐infected cells or cells treated with diABZI (Figure 5C, Panel 3) served as controls.

Altogether, these results suggested that the cGAS‐STING pathway is activated at late stages postinfection, leading to an IFN type I response.

**Figure 6:**
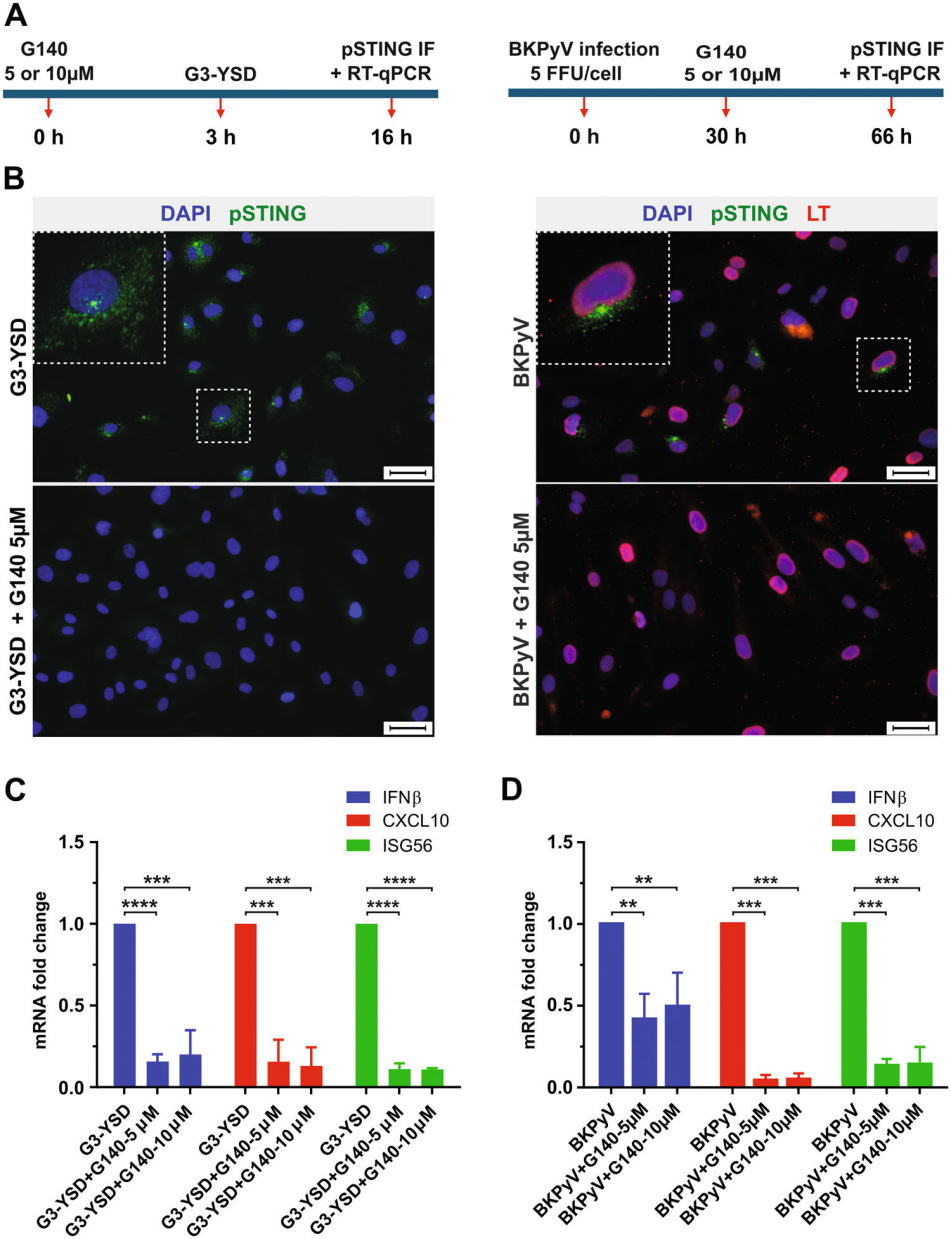
Blocking the ATP‐GTP active binding pocket in cGAS using G140 significantly inhibited the innate immune response of human bladder microvascular endothelial cells (HBMVECs) to BK polyomavirus (BKPyV) and the cGAS agonist G3‐YSD. HBMVECs were infected with BKPyV or transfected with G3‐YSD and treated with the cGAS inhibitor G‐140 (5 and/or 10 µM) at the indicated times. As control, cells infected with BKPyV or transfected with G3‐YSD were used. (A) A schematic diagram of the experiment. (B) Cells were fixed and stained with antibodies against phospho‐STING (green) and LT (red) while DNA was stained with DAPI (blue). Scale bars correspond to 50 µm. Selected cells (dotted white boxes) are magnified and displayed (bigger dotted white boxes). (C, D) HBMVECs were harvested at the indicated times for RT‐qPCR. For each sample, the mRNA levels of *IFNB*, *CXCL10*, and *ISG56* were normalized to those of *GAPDH*, and the fold increase was calculated by comparing each sample to the mock condition. Data were further analyzed by calculating the fold decrease of transcript levels in activated (by cGAS or infected), treated with G140 cells, compared with those activated but not‐treated cells. The data correspond to the means ± SD of three independent experiments. Statistics were calculated using GraphPad Prism software version 10. Probability levels are indicated by **p ≤ 0.01 or ***p ˂ 0.0001, ****Student's *t* test.

Given the challenges associated with the transfection of primary cells, it was not possible to knock down cGAS. Therefore, to validate the observations regarding the role of this DNA sensor in the IFN response, the cGAS inhibitor, G140, was employed instead. G140 occupies the ATP‐ and GTP‐binding active site in cGAS, effectively blocking cGAMP production [41]. The G140 inhibitor did not induce toxicity in the HBMVECs (data not shown). The experimental conditions were evaluated in pilot experiments (Figure 6A). For the experiments, the cGAS agonist, G3‐YSD, served as a control. First, STING phosphorylation was monitored by immunofluorescence (Figure 6B). Treatment with G140 significantly reduced STING phosphorylation in cells exposed to G3‐YSD and in BKPyV‐infected cells, the IFN type I response (transcripts levels of *IFNB*, *CXCL10*, *ISG56*) was also reduced in the presence of the inhibitor in both G3‐YSD‐treated and BKPyV‐infected cells (Figures 6C,D).

These results highlight the critical role of the cGAS‐STING pathway in the IFN response of HBMVECs to BKPyV infection.

**Figure 7:**
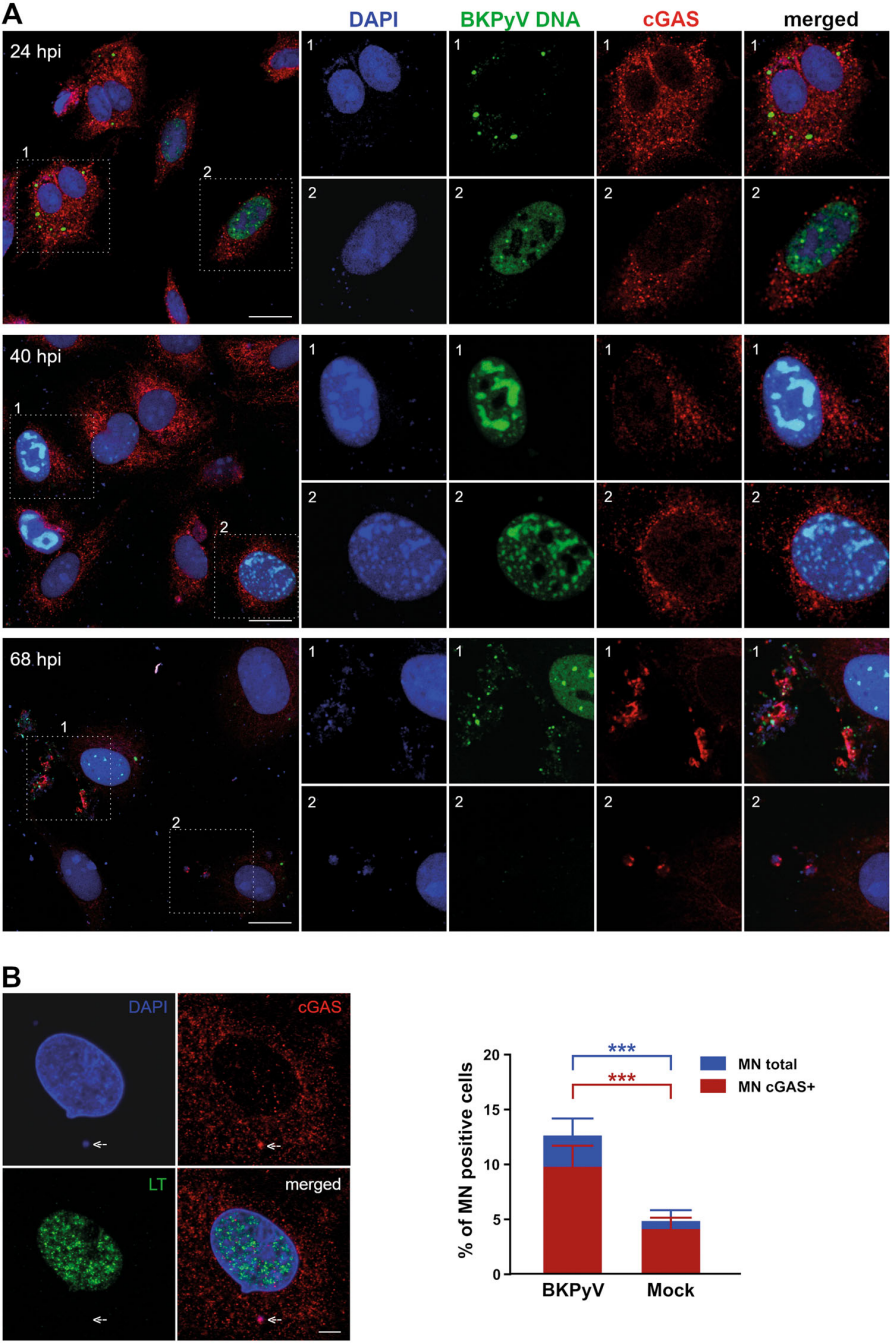
Viral and cellular DNA colocalize with cGAS during the late stage of BK polyomavirus (BKPyV) infection. (A) Confocal sections of selected fields of human bladder microvascular endothelial cells (HBMVECs) infected with BKPyV at MOI 10 FFU/cell and fixed at the indicated times are shown. Viral DNA (green) was visualized by fluorescence in situ hybridization (FISH); cGAS (red) was stained with a specific antibody and cellular DNA (blue) was detected with DAPI. Regions of interest inside dotted white boxes are magnified and displayed in separate channels. Scale bar = 20 μm. (B) Mock‐ or BKPyV‐infected (68 hpi) HBMVECs were fixed, immunolabeled for cGAS (red) and LT (green) proteins, counterstained with DAPI (blue; DNA), and assessed for the presence of micronuclei by fluorescence microscopy. A confocal section of a representative infected cell containing a micronucleus (arrow) is shown on the left. Scale bar = 5 μm. The percentage of cells containing micronuclei (MN total) and the percentage of cells containing micronuclei positive for cGAS (MN cGAS+) are shown in the graph (right). The data correspond to the mean values of three independent experiments (at least 450 cells were examined in each experiment). ***p ˂ 0.001, Student's *t* test.

Finally, putative colocalization of the BKPyV genomes with cGAS was assessed at different times postinfection by fluorescence in situ hybridization (FISH) assay. For this, primary HBMVECs were infected with BKPyV and fixed on coverslips at 24, 40, and 68 hpi (Figure 7A).

The cGAS signal was dispersed throughout the cytosol in uninfected cells (data not shown) as well as in infected cells at 24 and 40 hpi (Figure 7A). At 62 (Supporting Information S2: Figure 2) and 68 hpi, sporadic clusters of viral or host DNA and cGAS could be seen in the cytosol (Figure 7A). In addition, the number of micronuclei loaded with cGAS was quantified in both, mock‐infected and infected cells at 68 hpi. The number of micronuclei increases by approximately 10% in infected cells relative to the control (Figure 7B).

These results suggested that, at late stages of infection, cGAS in the cytosol senses the presence of viral and cellular DNA leaked from the nucleus and induces the activation of STING and the production of IFN and cytokines.

## 3 Discussion and Conclusions

### 3.1 HBMVECs a Good Model to Study BKPyV

BKPyV is a virus that establishes a persistent infection primarily in the urinary tract and can occasionally be detected in the urine of healthy individuals. In a recent study, An et al. suggested that bladder microvascular endothelial cells might serve as potential viral reservoirs [33]. However, although the HBMVECs used in this study were obtained from anonymous donors, no evidence of BKPyV infection was found. In particular, no BKPyV proteins (LT or VP1), LT transcripts, or viral genomes were detected in these cells (primers are described in Supporting Information S3: Methods), suggesting that the cells used in this study may have been derived from hosts that do not carry the virus. Alternatively, these cells may contain BKPyV archetypal genomes that are below the detection limit and do not replicate in tissue culture [8]. Finally, while HBMVECs represent a suitable model for studying responses to BKPyV in reservoir cells, they may not be the primary reservoir for the virus in vivo.

### 3.2 BKPyV Replication in HBMVECs Results in the Production of TRSs and Vacuolization

The BKPyV life cycle in HBMVECs follows a similar pattern and kinetics to that of the RPTECs. In RPTECs, virus release starts at low levels at 48 hpi and continues progressively for 4–5 days. Interestingly, there was also a modest increase in cell death in RPTECs starting at 72 hpi and continuing for 2 more days [18, 39]. The internalization of BKPyV into monopinocytic vesicles and virus sorting to late endosomes has been previously described for RPTECs [12, 18]; however, the presence of BKPyV in TRSs, which was observed at early and late stages of infection (in this study), has not been documented yet as a feature of BKPyV infection in tissue culture, although it has been observed in biopsies of patients with BKPyV‐related nephropathy [42, 43].

TRSs are commonly seen in viral infections and are also induced by stress stimuli such as IFNs [44]. While these structures were first described in SV40‐infected cells in 1989 [45], their role in SV40 infection has only recently been investigated. Bagchi et al. showed that SV40 hijacks the activity of lunapark, an ER membrane protein known to stabilize multitubular three‐way ER junctions, to form sites that allow for virus escape to the cytosol [46]. Meanwhile, the formation of vacuole‐like vesicles and neutral lipid droplets was also observed in the present study. Recent studies have shown a clear link between lipid metabolism and ER stress [47]. Vacuolization has also been documented in biopsies of patients with BKPyV‐associated nephropathy, while RNA sequencing analysis revealed ER stress responses in these tissues [48]. The presence of TRSs and lipid droplets likely reflect ER remodeling and stress. These processes may affect the life cycle of the virus and influence cellular fate. Further investigations are needed to elucidate the nature and role of these structures in BKPyV infection.

### 3.3 The IFN Response at Late Stages of Infection May Facilitate BKPyV Persistence

Regarding IFN responses, the data presented here suggest that moderate activation of the innate immune response at a late stage of infection is a unique virus strategy that may support viral persistence. For BKPyV, persistence means that the virus can continue to replicate within the host, but at low levels. This approach ensures that some virus is produced, while simultaneously triggering an antiviral state in cells. This antiviral state prevents acute infection in the short term but could allow periodic virus production in the long term. Interestingly, the cGAS‐mediated response to the virus or G3‐YSD appears moderate compared to responses to the STING agonist diABZI, possibly because cGAS activation is precisely modulated in these cells. However, it is also possible that viral proteins interfere with the IFN responses at one or more levels. In fact, agnoprotein has been shown to disrupt the mitochondria in RPTECs [32]. Thus, through its affinity for membranes, agnoprotein could also disturb the ER organization and therefore the STING signaling. Functional studies will be necessary to elucidate the regulatory mechanism of the activation of cGAS‐STING pathway in HBMVECs.

The findings of this study suggest that cGAS activation in the cytosol during the late phase of infection is induced by the leakage of viral and host DNA from the nucleus. This leakage is a result of genotoxic stress and a situation where viral replication centers and host DNA are being pushed out toward the periphery of the nucleus by loads of virion progeny, leading to nuclear membrane perturbation and extensive (active) virion release from the cell nucleus to the cytoplasm. An important subject of a future study will be to describe the mechanism of virus exit from the host cells and to confirm, whether mitochondrial DNA release can also contribute to the cGAS activation of the IFN response.

## 4 Materials and Methods

### 4.1 Cell Lines and Virus Preparation

Primary HBMVECs (P10989; Innoprot) and RPTECs (RPTECs/TERT1 cells; Evercyte, CHT‐003‐0002) were cultured following the supplier's instructions (using ProxUp2 medium [Evercyte] or P60104 medium [Innoprot], respectively). BKPyV strain Dunlop (GenBank accession no. KP412983) was prepared from the pBKV (34‐2) plasmid containing the complete BKV genome (ATCC; 45025). The BKPyV genome was excised from the plasmid with BamHI endonuclease and the resultant linear fragments were diluted to a concentration of 5 μg/mL and ligated. The ligated fragments were transfected into RPTECs/TERT1 cells by electroporation in a Nucleofector device (Lonza, Basel, Switzerland). The cells were cultured until a substantial cytopathic effect was observed, following which the medium containing the viral particles was collected and used as inoculum for further virus propagation. RPTECs/TERT1 cells were infected with virus inoculum from transfected cells (MOI = 0.01 FFUs/cell) and incubated until a prominent cytopathic effect was visible. The virus was isolated and purified by CsCl density gradient centrifugation [49]. Viral titers were quantified using HBMVECs.

### 4.2 Electron Transmission Microscopy

Cells grown on glass coverslips were fixed in 4% formaldehyde and 2% glutaraldehyde in 0.05 M sodium cacodylate buffer. The cells were then post‐fixed in 1% osmium tetroxide for 1 h on ice, washed in distilled water, contrasted with 0.5% uranyl acetate, dehydrated using a graded ethanol series and propylene oxide, infiltrated with and flat‐embedded in epoxy resin (EMBED 812 Kit; EMS #14120), and polymerized at 60°C for 48 h. Ultrathin sections (60 nm) were cut from the block face parallel to the cell monolayer (horizontal sections) using a LEICA EM UC7 ultramicrotome equipped with a diamond knife (DIATOME), collected on formvar/carbon‐coated copper grids, and post‐contrasted with lead citrate. Imaging data were collected on a JEM‐2100 Plus Transmission Electron Microscope (JEOL; operated at 200 kV) equipped with a TVIPS TemCam–XF416 4 K CMOS camera.

### 4.3 SDS/PAGE and Western Blot Analysis

Cell lysates were prepared in RIPA lysis buffer supplemented with a protease inhibitor cocktail (Complete Mini EDTA free) and PhosSTOP (both from Roche). The proteins were resuspended in Laemmli buffer and separated by 10% SDS/PAGE. The gels were blotted onto nitrocellulose membranes and immunoassayed using the indicated antibodies. The bands were analyzed by chemiluminescence using the Amersham Imager 600 (GE Healthcare, Chicago, IL, USA).

### 4.4 Reverse Transcription‐Quantitative PCR (RT‐qPCR)

All the reagents and kits used for RT‐qPCR are listed in Table 1. In brief, total RNA was extracted from cells and its concentration and purity were measured using a Nanodrop spectrophotometer (Thermo Fisher Scientific, Waltham, MA, USA). Following reverse transcription, the cDNA was PCR‐amplified using a LightCycler 480 Instrument II (Roche) and subsequently quantified.

**Table 1:** Reagents.

Antibodies
Rabbit(E9A9K) Phospho‐STING ‐Ser366 (Cell signaling Technology)
Rabbit (D2P2F) STING (Cell signaling Technology)
Rabbit polyclonal anti‐GAPDH (Merck)
Rabbit cGAS (E5V3W) mAb(Cell signaling Technology)
Rabbit NF‐kB p65 (D14E12)XP (Cell signaling Technology)
Rabbit IRF3 (D83B9) (Cell signaling Technology)
Mouse SV40 T‐antigen–antibody (PAb416) (Abcam)
Rabbit BK virus VP1 pAb (prepared in our laboratory)
The secondary antibodies used were goat anti‐rabbit conjugated with peroxidase (Bio‐Rad), donkey anti‐rabbit conjugated with AlexaFluor 488 or 546, goat anti‐mouse conjugated with AlexaFluor 488 or 546 (Thermo Fisher Scientific)
**Primers**
IFN β: 5′‐TGGCACAACAGGTAGTAGGCG‐3′ and 5′‐TGGAGAAGCACAACAGGAGAGC‐3′
ISG56: 5′‐CTAAGCAAAACCCTGCAGAAC‐3′ and 5′‐TCAGGCATTCATCGTCATC‐3′
ISG15: 5′‐ACGAACCTCTGAGCATCCTGG‐3′ and 5′‐AAGGTCAGCCAGAACAGGTCG‐3’
CXCL10: 5′‐GTGGCATTCAAGGAGTACCTC‐3′ and 5′‐GCCTTCGATTCTGGATTCAGACA‐3’
GAPDH: 5′‐CACATCGCTGAGACACCATG‐3′ and 5′‐TGACGGTGCCATGGAATTTG‐3’
**Dyes and chemicals**
Hoechst 33342 (Thermo Fisher Scientific)
DAPI (Thermo Fisher Scientific)
HCS LipidTOX™ Red Neutral Lipid Stain (Thermo Fisher Scientific)
G140‐G‐140 Human cGAS inhibitor (Invivogen)
G3‐YSD ‐Y‐form DNA – cGAS agonist (Invivogen)
Poly(I)‐Poly(C) – (Cytiva)
diABZI‐Non‐nucleotide‐based STING agonist (trihydrochloride form) – (Invivogen)
Turbofect (Thermo Fisher Scientific)
**Kits**
Amaxa Kit V (Lonza)
LunaScript® RT SuperMix Kit (New England Biolabs)
NucleoSpin RNA (Macherey Nagel)
KiCqStart® SYBR® Green qPCR ReadyMix (Merck)

### 4.5 cGAMP Quantification

cGAMP was quantified using a 2′3′‐cGAMP ELISA Kit (Arbor Assays, Ann Arbor, MI, USA) following the manufacturer's protocol. The optical density (OD) was measured at 450 nm using an ELISA microplate reader (Tecan, Männedorf, Switzerland). The data were processed using the Four Parameter logistic (4PL) regression model.

### 4.6 Cell Transfection and Stimulation With Inducers of IFN

A total of 1.5 × 10^5^ cells were incubated with 0.1 μg of poly(I:C) for 16 h. cGAMP agonist transfection was performed with TurboFect. For experiments on 6‐cm plates, 2 × 10^6^ cells were transfected with 4 μg of the G3‐YSD, while for coverslips, 1.5 × 10^5^ cells were transfected with 0.3 μg of G3‐YSD. Following transfection, the cells were incubated for 16 h. For STING agonist treatment, cells were incubated for 3–6 h in a medium containing diABZI at a concentration of 2 µM (for the reagents used, see Table 1).

### 4.7 Virus Infection

On the day of infection, cells at approximately 80% confluence were washed with serum‐free DMEM and incubated with BKPyV diluted in serum‐free medium for 1.5 h at 37°C. After virus adsorption, the cells were washed to remove the unbound virus, and incubated in a medium until the desired time point of an assay.

### 4.8 Immunofluorescence Staining

Cells grown on coverslips were fixed in 4% paraformaldehyde and processed as previously reported [35]. Images were obtained using an LSM 880NLO confocal microscope (Carl Zeiss, Oberkochen, Germany), a Leica DMi8 WF microscope, and an Olympus IX73 Inverted Microscope. The analysis of data Figure 1B–D is described in Supporting Information S3: Methods.

### 4.9 FISH

FISH was performed in combination with immunofluorescence following the protocol described before [35]. The probe was generated by nick translation of the pBKV (34‐2) plasmid using the Atto488 NT Labelling Kit (Jena Bioscience).

### 4.10 Antibodies, Dyes, and Primers

Reagents not described in the text of this article are listed in Table 1.

## Author Contributions

S.H., K.B., B.R., L.H., and D.L. performed experiments and analyzed data. S.H.K.B. and B.R. designed experiments. S.H., D.L., K.B., and J.F. wrote the manuscript. All authors read and approved the final manuscript.

## Ethics Statement

The authors have nothing to report.

## Consent

The authors have nothing to report.

## Conflicts of Interests

The authors declare no conflicts of interest.

## Supporting information

Supporting information.

Supporting information.

Supporting information.

## Data Availability

The data that support the findings of this study are available on request from the corresponding author. The data are not publicly available due to privacy or ethical restrictions. The data that support the findings of this study are available upon reasonable request.
