# Supplementary material for: BK Polyomavirus Infection of Bladder Microvascular Endothelial Cells Leads to the Activation of the cGAS‐STING Pathway
Source: J Med Virol. 2024 Nov 2;96(11):e70038. doi: 10.1002/jmv.70038 (PMC11600483; doi:10.1002/jmv.70038)
Supplement: Supplementary file 1 — Supporting information. [file JMV-96-e70038-s001.docx]

**
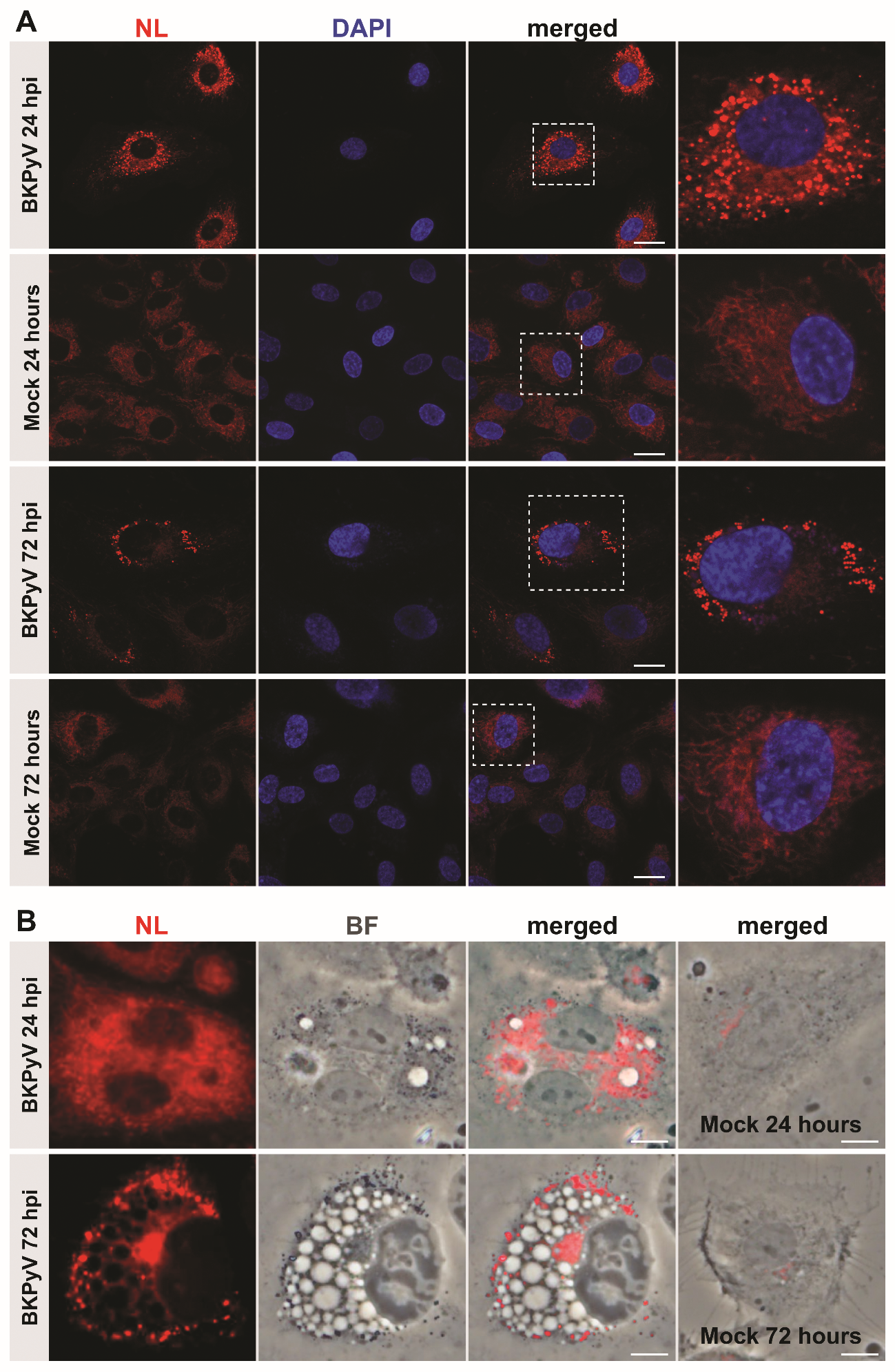
**

**Supplemental Figure 1. Formation of neutral lipid droplets and vacuole-like vesicles is induced during BK Polyomavirus (BKPyV) infection**. Mock or BKPyV-infected (MOI 10 FFU/cell) human bladder microvascular endothelial cells (HBMVECs) were fixed at the indicated times and neutral lipids (NL) were stained using HCS LipidTOX™ Red Neutral Lipid Stain (Thermo Fisher Scientific) according to the manufacturer instuctions. (A) Confocal sections of selected fields are shown. NL are shown in red. The DNA was stained with DAPI (blue). Scale bars correspond to 20 μm. Representative cells inside white boxes were magnified and shown in the right panel (B) The presence of vacuoles-like vesicles (bright field - BF) was analyzed by widefield microscopy. Neutral Lipids are shown in red. Scale bars correspond to 20 μm.
