# Supplementary material for: BK Polyomavirus Infection of Bladder Microvascular Endothelial Cells Leads to the Activation of the cGAS‐STING Pathway
Source: J Med Virol. 2024 Nov 2;96(11):e70038. doi: 10.1002/jmv.70038 (PMC11600483; doi:10.1002/jmv.70038)
Supplement: Supplementary file 2 — Supporting information. [file JMV-96-e70038-s003.docx]

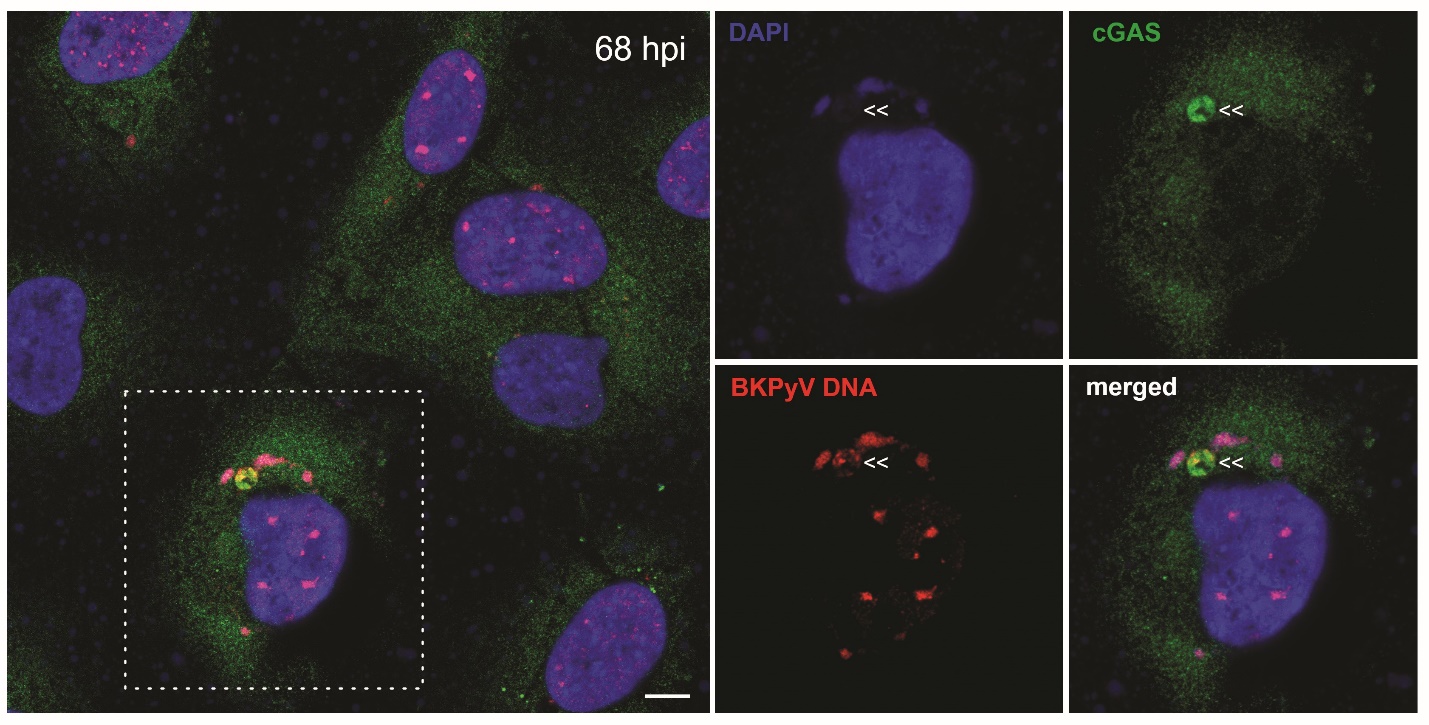


**Supplemental Figure 2. Viral DNA colocalizes with cGAS.** Representative confocal section of human bladder microvascular endothelial cells (HBMVECs) infected with BK polyomavirus (BKPyV) at a multiplicity of infection (MOI) of 10 fluorescent focus-forming units (FFUs) per cell. Cells were fixed at 62 hours post-infection (hpi). Viral DNA (red) was visualized by fluorescence *in situ* hybridization (FISH); cGAS (green) was stained with a specific antibody and cellular DNA (blue) was detected with DAPI. FISH was performed using a biotinylated BKPyV DNA probe according to a previously described protocol (Ryabchenko et al., 2021), with minor modifications. Biotin detection was performed using Alexa Fluor 594-conjugated streptavidin (Invitrogen). The region of interest inside the dotted white box is magnified and displayed in separate channels. The region of viral DNA and cGAS colocalization is indicated by an arrow. Scale bar = 10 μm.

Reference:

Ryabchenko, B., et al., *Immune sensing of mouse polyomavirus DNA by p204 and cGAS DNA sensors.* FEBS J, 2021. **288**(20): p. 5964-5985.
