# Supplementary material for: BK Polyomavirus Infection of Bladder Microvascular Endothelial Cells Leads to the Activation of the cGAS‐STING Pathway
Source: J Med Virol. 2024 Nov 2;96(11):e70038. doi: 10.1002/jmv.70038 (PMC11600483; doi:10.1002/jmv.70038)
Supplement: Supplementary file 3 — Supporting information. [file JMV-96-e70038-s002.docx]

Image Acquisition and Processing

Image data underwent processing using a custom Python workflow employing the Cellpose library for nuclei segmentation (Stringer et al., 2021). Signal intensity within segmented nuclei was quantified as the mean intensity across the nucleus for each channel (DAPI, LT, VP1). The resulting data were structured for temporal analysis. **Data Availability:** All processing scripts are accessible on GitHub at [GitHub Repository](<https://github.com/IMCF-Biocev/Virus-Infection-Analysis-Workflow/tree/main>). Raw data and segmentation masks are also accessible via the OMERO server upon request.

Stringer, Carsen, et al. "Cellpose: a generalist algorithm for cellular segmentation." Nature methods 18.1 (2021): 100-106."

**Detection of genomes of BK polyomavirus**

We used qPCR with SYBR Green to detect BKPyV genomes and and two sets of primers amplifying the VP1 region. This sequence, relatively conserved among BKPyV types and isolates, is based on the manuscript of Hoffman et al.

Primer sequences:

BK Ib2 FW 5´-GGGCCTCTTTGTAAAGCTGATA-3´ (2317-2338),

BK Ib2 REV 5´-CTCCACTGTTGTGTTCCAGAG-3 (2385-2405), and

BK IV FW 5´-AGAATCCTTACCCAATTTCCTT-3´ (2453-2474),

BK IV REV 5´-ATAGGCTGCCCATCCAC-3 (2512-2528).

Numbers refer to the position on the reference BKPyV Dunlop strain (GenBank # V01108.1)

Reference:

Hoffman NG, Cook L, Atienza EE, Limaye AP, Jerome KR. Marked

variability of BK virus load measurement using quantitative real-time PCR

among commonly used assays. J Clin Microbiol (2008) 46(8):2671–80. doi:

10.1128/JCM.00258-08

**Detection of LT - BK polyomavirus transcripts**

We used qPCR with SYBR Green to detect LT transcripts using the primers:

BK LT F 5´-GAGTAGCTCAGAGGTGCCAACC-3´ (4911-4923+4566-4558)

BK LT R 5´-CATCACTGGCAAACATATCTTCATGGC -3´ (4464-4490)

Numbers refer to the position on the reference BKPyV Dunlop strain (GenBank # V01108.)
